# Reactive control processes contributing to residual switch cost and mixing cost across the adult lifespan

**DOI:** 10.3389/fpsyg.2014.00383

**Published:** 2014-04-30

**Authors:** Lisa R. Whitson, Frini Karayanidis, Ross Fulham, Alexander Provost, Patricia T. Michie, Andrew Heathcote, Shulan Hsieh

**Affiliations:** ^1^Functional Neuroimaging Laboratory, School of Psychology, University of NewcastleCallaghan, NSW, Australia; ^2^Centre for Translational Neuroscience and Mental Health Research, The University of NewcastleCallaghan, NSW, Australia; ^3^Hunter Medical Research Institute, New Lambton HeightsNSW, Australia; ^4^Department of Psychology and Institute of Allied Health Sciences, National Cheng Kung UniversityTainan City, Taiwan

**Keywords:** cognitive control, aging, lifespan, ERP, evidence accumulation models

## Abstract

In task-switching paradigms, performance is better when repeating the same task than when alternating between tasks (switch cost) and when repeating a task alone rather than intermixed with another task (mixing cost). These costs remain even after extensive practice and when task cues enable advanced preparation (residual costs). Moreover, residual reaction time mixing cost has been consistently shown to increase with age. Residual switch and mixing costs modulate the amplitude of the stimulus-locked P3b. This mixing effect is disproportionately larger in older adults who also prepare more for and respond more cautiously on these “mixed” repeat trials (Karayanidis et al., 2011). In this paper, we analyze stimulus-locked and response-locked P3 and lateralized readiness potentials to identify whether residual switch and mixing cost arise from the need to control interference at the level of stimulus processing or response processing. Residual mixing cost was associated with control of stimulus-level interference, whereas residual switch cost was also associated with a delay in response selection. In older adults, the disproportionate increase in mixing cost was associated with greater interference at the level of decision-response mapping and response programming for repeat trials in mixed-task blocks. These findings suggest that older adults strategically recruit greater proactive and reactive control to overcome increased susceptibility to post-stimulus interference. This interpretation is consistent with recruitment of compensatory strategies to compensate for reduced repetition benefit rather than an overall decline on cognitive flexibility.

## INTRODUCTION

Cognitive control encompasses proactive (e.g., anticipatory engagement and maintenance of task goals) and reactive control processes (e.g., conflict monitoring and interference resolution) to adjust and maintain goal-directed behavior (Braver, 2012). The task-switching paradigm differentiates between proactive and reactive control processes involved in shifting between task rules (for review see Kiesel et al., 2010) and has been used to examine how these processes contribute to age-related cognitive decline. Compared to young adults, older adults benefit less from task repetition (for review see Kray and Ferdinand, in press) and show greater post-stimulus interference for repeat trials (Karayanidis et al., 2011), suggesting a greater need to apply reactive control. In the present study, we apply novel analyses to the target-locked event-related potential (ERP) data presented in Karayanidis et al. (2011) in order to examine whether reactive control is applied to resolve interference at the level of stimulus or response processing, and whether this differs between young and old adults.

**FIGURE 1:**
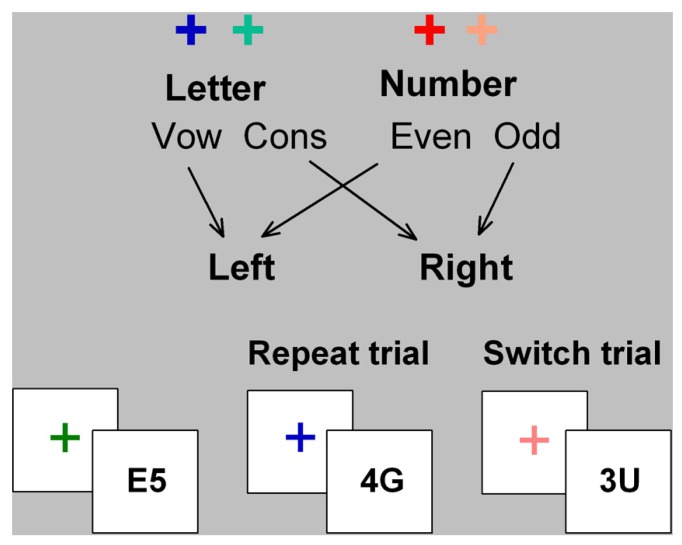
**Cued-trials task switching paradigm.** Letter and number classification tasks were consistently mapped to hot or cold color cues (cross) and to left and right hand responses. The stimulus consisted of a letter and a number that are incongruently mapped to response hand (i.e., on mapping shown here, if letter is a vowel, number will be odd, and *vice versa*). Cue color changed on every trial and validly signaled the relevant task for the upcoming stimulus. The cue was removed upon stimulus onset and the stimulus did not contain information about which task was relevant on that trial.

In cued-trials task-switching paradigms, participants alternate between two simple tasks using cues that validly indicate whether to switch or repeat tasks (**Figure 1**). In mixed-task blocks, *switch cost* is estimated as the difference in performance between *switch* trials and *mixed-repeat* trials (i.e., repeat trials in a mixed-task block) and represents the time taken to reconfigure to the new task-set and resolve interference from the old task-set. *Mixing cost* is estimated as the performance difference between mixed-repeat and *all-repeat trials* (i.e., trials in a single-task block) and is attributed to increased demands on working memory, greater task ambiguity, and/or failure to fully disengage the alternative task-set (e.g., Mayr, 2001; Meiran and Gotler, 2001)^1^. Both mixing cost and switch cost reduce as the cue-stimulus interval increases, indicating the engagement of proactive control processes. However, even with long preparation intervals, residual performance costs remain. While residual mixing and switch cost may arise partly from failure to prepare on some proportion of trials (De Jong, 2000), they are also modulated by stimulus-related and response-related parameters. Thus, even under prepared task conditions, reactive control processes may be activated to control stimulus-driven interference (for review see Kiesel et al., 2010).

Event-related potential studies using the task-switching paradigm have identified electrophysiological correlates of proactive and reactive control processes (for review see Karayanidis et al., 2010; Karayanidis and Jamadar, in press). Within the cue-stimulus interval, an early parietal “*mixing positivity*” is elicited for mixed-task relative to single-task blocks and a later parietal “*switch-positivity*” is elicited for switch relative to mixed-repeat trials in the mixed-task block. Stimulus-locked ERP waveforms derived from long preparation conditions show both mixing-related and switch-related modulation of frontocentral N2 and centroparietal P3b components that are related to conflict control and decision processes, respectively. Switch-related modulation of both N2 and P3b varies as a function of stimulus-driven interference (Karayanidis et al., 2003; Poulsen et al., 2005), is inversely related to reaction time (RT) and RT switch cost (e.g., Lavric et al., 2008), and is maintained even after substantial task practice (Karayanidis et al., 2003, 2011). These results are consistent with residual switch cost representing the recruitment of reactive control to overcome sustained post-stimulus interference from the previously relevant task-set (Karayanidis and Jamadar, in press). However, the relative contribution of stimulus-level and response-level interference to residual switch cost has not been systematically examined.

### AGE EFFECTS ON RESIDUAL MIXING AND SWITCH COSTS

Older adults show a robust increase in residual mixing cost (e.g., Kramer et al., 1999; Meiran et al., 2001; Kray, 2006), but age effects on residual switch cost are less consistent (e.g., Kray et al., 2002; Kray and Lindenberger, 2000; Kray, 2006; Hsieh and Wu, 2010). This variability is at least partly due to differential effects of task practice in young and old adults. Whitson et al. (2012) reported that early in task exposure, both residual mixing cost and residual switch cost were larger in old than young adults. After considerable task practice, old adults retained a larger residual mixing cost compared to young adults (see also Kray and Lindenberger, 2000; Cepeda et al., 2001), but did not differ in residual switch cost (see also Kramer et al., 1999; Buchler et al., 2008). Differential age effects on residual mixing and switch cost were explained by prolonged RT for mixed-repeat trials in older adults, suggesting that old adults processed mixed-repeat trials much like switch trials (Mayr, 2001). This interpretation was supported by age differences in preparation as evidenced by a larger and more prolonged mixing-positivity but a smaller switch-positivity in old than in young adults (Karayanidis et al., 2011).

This conclusion was also supported by evidence accumulation model analyses (e.g., Grasman et al., 2009). In mixed-task blocks, young adults adjusted response criterion on a trial-by-trial basis by setting a more conservative decision threshold on switch than on mixed-repeat trials (Karayanidis et al., 2009, 2011; Schmitz and Voss, 2012), whereas old adults maintained the same high criterion for both trial types (Karayanidis et al., 2011). Thus, old adults either have a preference for more conservative decision-making (e.g., Ratcliff et al., 2005) or find it difficult to flexibly adjust criterion across trials (Forstmann et al., 2011). Furthermore, age moderated the strength of the relationship between response criterion and both the mixing-positivity and the switch-positivity (Karayanidis et al., 2011), consistent with the notion that aging affects proactive control processes involved in the flexible adjustment of response criteria (Karayanidis et al., 2009; Mansfield et al., 2011).

Given converging evidence that old adults prepare for both mixed-repeat and switch trials, it is reasonable to expect that they will show a similar level of post-stimulus interference for these two trial types and hence *less* differentiation between stimulus-locked ERPs for mixed-repeat and switch trials than young adults. Yet Karayanidis et al. (2011) reported that old adults showed similar differentiation between switch and mixed-repeat trials as young adults, as well as much larger differentiation between mixed-repeat and all-repeat trials^2^. Thus, despite greater preparation for mixed-repeat trials, old adults showed greater difficulty selecting between or implementing different task-sets in the context of task ambiguity (see also Mayr, 2001; Kray et al., 2002).

### LOCUS OF RESIDUAL COSTS IN TASK-SWITCHING

The first aim of the present study is to examine the locus of residual switch cost and mixing cost. Specifically, we examine whether residual costs in highly practiced participants arise from post-stimulus interference at the level of stimulus or response processing. We also examine whether the age-related increase in residual mixing cost on both RT and the stimulus-locked P3b results from greater need for reactive control to resolve interference at the stimulus or response level.

In Karayanidis et al. (2011), participants completed a cued-trials task-switching paradigm with a long preparation interval (cue-stimulus interval 1000 ms) and with bivalent stimuli that were incongruently mapped to response hand (**Figure 1**). Post-stimulus interference could therefore arise at the level of stimulus processing (e.g., selection of the relevant stimulus feature and interference from the irrelevant stimulus feature) as well as response processing (e.g., selection of the relevant response and interference from the alternative stimulus-response mapping). In the present study, we apply new analyses to the stimulus-locked ERP waveforms from Karayanidis et al. (2011) in order to differentiate between stimulus-level and response-level influences on post-stimulus processing and residual costs in young and old adults.

#### Stimulus-locked vs. response-locked P3

In stimulus-locked ERP waveforms, the P3b is associated with decision-related processes, with strong evidence that P3b latency is delayed and P3b amplitude is reduced for more difficult decisions (e.g., Donchin et al., 1978; Polich, 1987). While P3b is typically linked to stimulus-driven processes related to decision-making (e.g., Kutas et al., 1977; Donchin and Coles, 1988), there is also evidence that P3b is affected by tactical post-decision processes associated with response selection, such as mapping a decision to a response (Verleger et al., 2005; see also Rösler et al., 1985). The effects of pre-decision and post-decision processes on the P3b can be dissociated by comparing ERP waveforms time-locked to stimulus vs. response onset. Manipulations that affect sensory and perceptual processes contributing to a decision only modulate stimulus-locked ERPs (Mordkoff and Gianaros, 2000), whereas manipulations that impact on post-decision processes (i.e., decision-to-response mapping, response activation) will be equally evident in both stimulus-locked and response-locked waveforms.

In this study, we apply this novel approach to distinguish between stimulus-level and response-level interference contributing to residual performance cost in task-switching and how these processes are modulated by aging. Specifically, we argue that, if stimulus-locked P3 (sP3) differences between trial types are eliminated in response-locked P3 (rP3) waveforms, they are likely to reflect the engagement of reactive control processes to deal with differential stimulus-level interference affecting the efficiency of stimulus encoding and/or stimulus evaluation. Alternatively, if stimulus-locked trial type effects are also evident in rP3 waveforms, they are likely to arise from response-level interference, affecting post-decision processes such as decision-to-response mapping and response programming.

#### Stimulus vs. response-locked LRP

Post-stimulus decision processes can also be studied using the lateralized readiness potential (LRP), which arises from differential activation over the motor cortex contralateral to the responding hand (De Jong et al., 1988; Gratton et al., 1988; Coles, 1989, for review see Smulders and Miller, 2012). The onset latency of the stimulus-locked LRP (sLRP) is the interval between stimulus onset and sLRP onset, and indexes the duration of sensory and perceptual processes leading up to response selection (Masaki et al., 2004). The response-locked LRP (rLRP) is time-locked to response onset and its duration (i.e., the interval between rLRP onset and the response itself) reflects the duration of motoric processes that occur after response selection, such as response programming and execution (Osman et al., 1995; Leuthold and Jentzsch, 2002). Hence, delayed sLRP onset can result from slower stimulus encoding and/or a delayed decision due to stimulus-level interference, whereas longer rLRP duration (i.e., a longer interval between rLRP onset and response) results from slower response programming due to response-level interference.

In this study, we analyze sLRP and rLRP components to help identify the locus of residual mixing and switch costs in task-switching in young adults and the increase in post-stimulus interference for mixed-repeat trials in old adults. Specifically, we examine whether delays in response selection and/or response programming contribute to residual mixing and switch cost in highly practiced young adults, and whether these mechanisms can account for increased residual RT mixing cost in old adults. Moreover, by comparing age effects on stimulus-locked and rP3 and LRP, we address the key question of whether the residual mixing effect on P3b amplitude in old adults (Karayanidis et al., 2011) is related to greater post-stimulus interference at the level of stimulus or response processes.

In task-switching, young adults show delayed sLRP onset, as well as earlier rLRP onset for mixed-repeat relative to all-repeat trials (Ruge et al., 2006). sLRP onset latency is also delayed for switch compared to mixed-repeat trials even with long preparation intervals (e.g., Hsieh and Yu, 2003; Hsieh and Liu, 2005; Hsieh and Chen, 2007; Umebayashi and Okita, 2008), whereas rLRP duration does not differ between mixed-repeat and switch trials (e.g., Hsieh and Yu, 2003; Hsieh and Chen, 2006, 2007; but see Hsieh and Liu, 2005). Hence, residual mixing cost is associated with both stimulus-level interference affecting response selection and response-level interference affecting response programming. In contrast, residual switch cost is associated with delays in response selection but not response programming.

To date, no study has used LRPs to examine the locus of age-related changes in task-switching performance. An age-related delay in sLRP onset latency has been reported on simple perceptual tasks (e.g., Ball and Sekuler, 1986; Van der Lubbe and Verleger, 2002) and interference tasks (Hsieh et al., 2011), but not on choice RT (Yordanova et al., 2004) and motion detection tasks (Roggeveen et al., 2007). Yet, on these more complex tasks, old adults show longer rLRP duration than young adults, suggesting slowed response programming.

### DECISION AND NON-DECISION PROCESSES CONTRIBUTING TO RESIDUAL COSTS IN TASK-SWITCHING

The second aim of this study is to examine the relationship between stimulus-related and response-related ERP measures and decision model parameters in order to identify the mechanisms that contribute to residual mixing cost and switch cost and their modulation with age.

Evidence accumulation models differentiate between decision and non-decision processes. The decision process is characterized by the drift rate (*v*, the rate of accumulating evidence favoring one or the other choice) and the response criterion (*a*, the threshold of evidence needed to make a decision). The process of evidence accumulation is conceptualized as beginning after stimulus encoding (e.g., a letter stimulus is identified before evidence toward a *vowel* or *consonant* boundary can begin to accumulate). The decision is derived when the evidence crosses either the *vowel* or the *consonant* boundary. Response selection, or the choice of the correct response effector based on the decision-to-response mapping (i.e., *vowel* is mapped to “*left*” hand, so press the left button), is also not part of the decision process. Rather, in two-choice decision tasks, stimulus encoding and response selection, as well as response programming, are incorporated within the non-decision parameter (*Ter*, i.e., time for encoding and responding) and cannot be independently measured. So, drift rate and criterion characterize the decision process but not the stimulus-related processes occurring before the onset of evidence accumulation or the response-related processes occurring after the decision. In task-switching paradigms, Ter also encompasses task-set shifting processes that have not been completed before stimulus encoding (Karayanidis et al., 2009; Schmitz and Voss, 2012) either because the preparation interval is too brief or because of failure to engage preparation on some proportion of trials (De Jong, 2000). It is important to note that this distinction between decision and non-decision processes does not neatly correspond to the ERP components outlined above, making it tricky to reconcile these different perspectives on decision-making processes.

**FIGURE 2:**
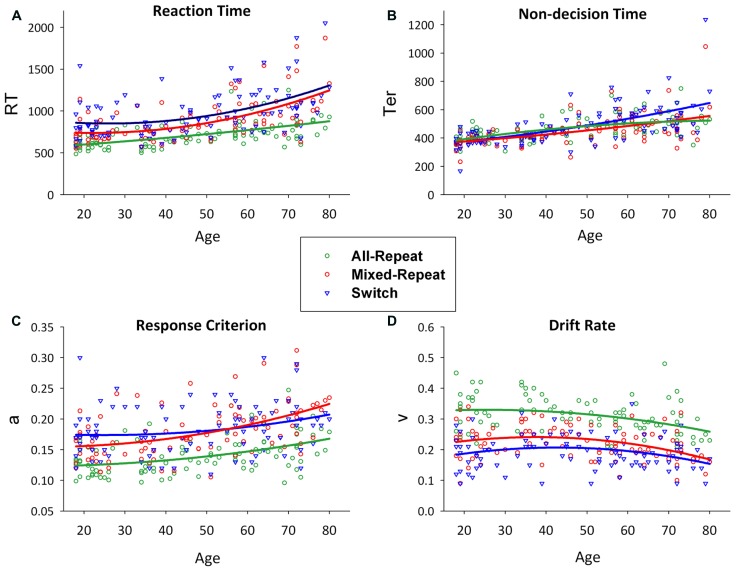
**Scatterplots of age against reaction time (A) and model parameters (non-decision time [B], response criterion [C] and drift rate [D]) for all-repeat (green), mixed-repeat (red) and switch (blue) trials.** Mixing cost is shown as the difference between green and red lines, and switch cost as the difference between red and blue lines. Adapted from (Karayanidis et al., 2011).

In young adults, behavioral mixing cost and switch cost derive most consistently from modulation of decision-related variables (i.e., relative increase of criterion and drift rate from all-repeat to mixed-repeat to switch trials), whereas switch-related modulation of non-decision processes varies with task parameters (Karayanidis et al., 2009, 2011; Schmitz and Voss, 2012). In Karayanidis et al. (2011), the age-related increase in RT mixing cost was due to a greater rate of increase in response criterion for mixed-repeat than all-repeat trials (see **Figure 2**). The small *reduction* in RT switch cost in older adults, despite an overall increase in non-decision time for switch trials (**Figure 2**; see also Madden et al., 2009), was due to the disproportionate increase in response criterion for mixed-repeat relative to switch trials and disproportionate reduction in switch cost on drift rate. Karayanidis et al. (2011) concluded that differential age effects on criterion for mixed-repeat trials and on both decision and non-decision parameters for switch trials may help explain the robust age effect on mixing cost but inconsistent effect on switch cost.

Karayanidis et al. (2011) showed that, when controlling for age, stimulus-locked P3b amplitude is inversely related with all three latent variables. However, the relative contribution of stimulus-level and response-related decision processes to this relationship could not be determined. In the present study, we dissect this relationship by combining stimulus-level and response-level analyses of the ERP and LRP waveforms with measures of decision and non-decision processes derived from evidence accumulation models to test hypotheses regarding the nature of residual costs in task-switching.

We assume that since highly practiced participants can efficiently prepare for switch trials (as evidenced by small residual switch cost and large cue-locked switch-positivity; Karayanidis et al., 2011), there is likely to be little contribution from cue processing or task-set shifting on non-decision time (i.e., Ter). Rather, individual variability in Ter is likely to represent differences in stimulus encoding and/or response processes that do not directly contribute to the decision. Specifically, we hypothesize that if the relationship between Ter and P3b (Karayanidis et al., 2011) is related to variability in response processes, Ter will be more strongly correlated with rP3 than with sP3, and any relationship between Ter and sP3 will be eliminated when controlling for rP3. In contrast, if the Ter and P3b relationship is related to variability in stimulus encoding, Ter and rP3 will not be correlated and/or the relationship between Ter and stimulus-locked P3b will be maintained when controlling for response-locked P3b. Variability in both drift rate and criterion are expected to be more strongly associated with the rP3 than the sP3, as the former is more tightly time-locked to the decision.

Stimulus-locked LRP onset latency represents lateralised activation toward a motor response, a process that cannot commence before the completion of stimulus encoding. If variability in the duration of stimulus encoding contributes to Ter, the latter will be significantly correlated with sLRP onset latency. sLRP onset latency is also expected to vary with the amount of information that must be accumulated before arriving at a decision (i.e., response criterion) and the rate at which this information is accumulated (i.e., drift rate). rLRP duration measures the post-decision processes that are associated with response programming. Therefore, rLRP duration is expected to be correlated with response processes that contribute to Ter and may delay response execution. In contrast, decision-related parameters (i.e., response criterion and drift rate) are not expected to be associated with rLRP duration.

## MATERIALS AND METHODS

### PARTICIPANTS

Ninety-five participants (18–80 years, mean age = 43±19.5 years, 32 male) with no neurological disorder, recent head injury, or color blindness contributed to this analysis. Participants had no prior experience with the task-switching experiment. Participants were allocated into four age groups based on breaks in mean RT across the age range (18–29 years: *n* = 25, 30–45 years: *n* = 20, 46–64 years: *n* = 20, 65–80 years: *n* = 30). Groups did not differ in distribution of gender, handedness or on full scale IQ (**Table 1**).

**Table 1:** Participant characteristics in each of the age groups (standard error in italics).

	18–29 years	30–45 years	46–59 years	60–79 years
*N*	25	20	20	30
Age	21.8 (*0.06*)	38.0 (*0.98*)	53.5 (*0.97*)	70.3 (*1.03*)
M:F ratio	9:16	4:16	6:14	13:17
FSIQ^a^	119 (*2.02*)	123 (*6.15*)	115 (*2.8*)	123 (*5.1*)

a Full scale IQ was estimated using the Wechsler Abbreviated Scale of Intelligence (Wechsler, 1999).

### TASKS AND PROCEDURE

A cued-trials task-switching paradigm was used (see **Figure 1**). Hot and cold cue colors were assigned to either a letter classification or a number classification task, respectively. Stimuli consisted of an incongruently mapped bivalent letter–number pair (e.g., A4 or 4A). Participants responded using left and right index fingers mapped to vowel/consonant or odd/even for letter and number tasks, respectively. Cue-task mapping and hand-task mapping were counterbalanced across participants. The stimulus was removed upon response or after 5000 ms. Errors were followed by immediate auditory feedback. Mean RT and error rate feedback was provided after each block of trials. On mixed-task blocks, switch probability was 50% with no more than four mixed-repeat or switch trials in succession.

Participants completed two practice sessions on the task-switching paradigm (448 trials/session) before the test session during which EEG was recorded (1344 trials). The test session included four single-task blocks (2 × 48 trials per task; CTI-1000 ms), and six mixed-task blocks (6 × 64 trials per block) for each CTI condition. CTI:RTI conditions were: 150:1400 1000:1400, and 150:750. As in Karayanidis et al. (2011), we only present ERP analyses from the long cue-target interval condition (192 trials per all-repeat, mixed-repeat and switch trials) which focuses on residual switch cost and mixing cost (e.g., stimulus-related and response-related processes under high preparation conditions).

### DIFFUSION MODEL PARAMETERS

Latent parameters for each trial type were estimated using the EZ2 diffusion model (Grasman et al., 2009) based on response accuracy, mean RT and variance of RT for correct decisions (see Karayanidis et al., 2011 for more details). In order to make mean and variance estimates robust, we based them on fits of the Ex-Gaussian distribution to correct RT deciles (Heathcote et al., 2002; see Wagenmakers et al., 2008, for a related approach to EZ estimation). We also based EZ2 estimates on the robust accuracy measure recommended by Snodgrass and Corwin (1988). In a few cases (<1%), *Ter* estimates were too small to be plausible (<100 ms). In such cases, we obtained parameter estimates by solving the EZ2 equations under the constraint that *Ter*>100 ms. Note that, without constraint, EZ2 parameters produce a perfectly accurate description of accuracy and correct RT mean and variance. Although this is not necessarily the case when a constraint is imposed, the effect of the constraint used on our data was negligible, so that the account of these measures remained essentially perfect.

### EEG RECORDING AND DATA ANALYSIS

EEG was recorded from 20 scalp sites using a Quikcap (10/20 system, nose reference, offline re-referencing to mastoids). EEG and vertical and horizontal EOG were continuously sampled at 500 Hz/channel on a Synamps II system (Neuroscan; impedance <5 kOhm) with a bandpass of 0.01–30 Hz using a 50 Hz notch filter. Vertical eyeblink artifact was corrected (Semlitsch et al., 1986) and sections of EEG contaminated with channel saturation or noise were marked for exclusion.

### STIMULUS-LOCKED AND RESPONSE-LOCKED ERP WAVEFORMS

For each trial type, a 1400 ms stimulus-locked epoch was extracted around stimulus onset (-200 to 1200 ms) and a 3000 ms response-locked epochs was extracted around response onset (-2000 to 1000 ms). Stimulus-locked and response-locked averages were baseline corrected using the same baseline (Verleger et al., 2005) which was set to –50 to 50 ms around stimulus onset. sP3 peak latency was measured using 50% fractional area latency over a 300–700 ms window and peak amplitude was the voltage at peak latency (Picton et al., 2000)^3^. rP3 peak amplitude was measured as voltage at peak latency using 50% fractional area latency from 400 ms before the response to 100 ms after the response.

### STIMULUS-LOCKED AND RESPONSE-LOCKED LRP

Stimulus-locked LRP and rLRP waveforms were extracted by averaging difference waveforms for left hand responses (C4–C3) and right hand responses (C3–C4; Coles, 1989). Waveforms were smoothed using a 25 ms moving average to reduce high frequency noise. sLRP onset latency was measured as 25% of peak amplitude over 200–600 ms^4^. rLRP onset latency was measured using 25% fractional area latency from 400 ms before the response to 100 ms after the response. Short sLRP onset latency indexes earlier onset of response selection, whereas longer rLRP duration (i.e., earlier onset of rLRP) indexes longer duration of response programming (i.e., longer interval between onset of response-related activation and actual response). Onset latency could not be estimated for sLRP in one participant and rLRP in another participant. They were treated as missing data in relevant analyses.

### DATA ANALYSIS

The first two trials of each run, trials associated with and immediately following an error, and trials associated with a response outside the pre-defined response window were excluded from all further analyses (200 ms – participant’s mean RT + 3 SD). The lower limit of 200 ms was selected as responses occurring prior to this are likely to represent reflexive/anticipatory responses or fast guessing not generated by engaging the appropriate task-set (Whelan, 2008). These criteria resulted in exclusion of an average of 3.35% of trials.

Age effects on ERP data were analyzed using two planned contrasts targeting *mixing cost* (all-repeat vs. mixed-repeat) and *switch cost* (mixed-repeat vs. switch) with centered age (*age*) and centered age squared (*age2*) as covariates. In addition, for comparability with prior studies, we directly compare the two extreme age groups (young vs. old) on switch cost and mixing cost contrasts using a mixed-design GLM.

### RELATIONSHIP BETWEEN ELECTROPHYSIOLOGICAL MEASURES, AGE, AND MODEL PARAMETERS

Pearson correlation coefficients were used to test hypotheses regarding the relationship between stimulus-locked and response-locked electrophysiological measures and model parameters. These correlations were run for each trial type individually, as well as for estimates of mixing cost and switch cost. Partial correlations were used to examine whether significant relationships between electrophysiological measures and model parameters survived when controlling for age. Significant correlations between model parameters and sP3 amplitude were also examined when also controlling for rP3 amplitude. For each variable (age and each model parameter), familywise error rate correction was applied (*a* = 0.05/10 = 0.005) separately for P3 and LRP measures. For each ERP measure, a linear regression was run with age and model parameters entered stepwise using the same significance level (*a* = 0.005). Correlation coefficients, the model that accounted for most of the variance and adjusted R^2^ are shown in **Tables 2** and **3**.

**Table 2:** ERP measures for stimulus-locked (sP3), response-locked P3 (rP3), stimulus-locked LRP (sLRP), and response-locked LRP (rLRP) for each age group.

			18–29 years		30–45 years		46–59 years		60–79 years	
			Mean	SE	Mean	SE	Mean	SE	Mean	SE
sP3	Pk latency	AR	490.45	*11.03*	512.65	*9.53*	514.66	*11.90*	532.99	*11.12*
		MR	527.03	*11.60*	539.38	*12.78*	510.14	*15.68*	517.76	*18.18*
		S	538.85	*13.23*	559.23	*12.81*	517.17	*17.90*	536.85	*17.85*
	Pk amplitude	AR	10.75	*0.86*	9.27	*1.02*	8.1	*0.87*	10.9	*0.82*
		MR	10.29	*0.96*	9.49	*1.04*	7.35	*0.68*	8.72	*0.60*
		S	9.17	*0.98*	8.03	*1.18*	6.25	*0.65*	6.9	*0.58*
rP3	Pk amplitude	AR	11.14	*1.00*	10.44	*1.03*	8.11	*0.80*	10.46	*0.78*
		MR	10.18	*0.98*	10.69	*1.33*	7.2	*0.88*	7.66	*0.64*
		S	9.25	*0.90*	10.05	*1.27*	7.62	*0.77*	7.42	*0.65*
sLRP	Pk amplitude	AR	-1.52	*0.15*	-2.09	*0.27*	-2.51	*0.26*	-2.41	*1.63*
		MR	-1.34	*0.18*	-1.98	*0.23*	-1.66	*0.16*	-1.78	*1.38*
		S	-1.1	*0.17*	-1.76	*0.21*	-1.3	*0.14*	-1.6	*1.39*
	Onset latency	AR	296.35	*15.17*	299.2	*10.85*	350.77	*11.24*	389.32	*73.10*
		MR	307.18	*16.33*	335.24	*14.61*	365.91	*15.16*	388.35	*53.04*
		S	350.18	*18.98*	313.08	*14.03*	378.33	*19.51*	364.06	*92.13*
rLRP	Onset latency	AR	-213.58	*10.68*	-251.7	*16.87*	-281.8	*12.48*	-289.03	*18.36*
		MR	-237.17	*13.63*	-289.3	*35.17*	-323.6	*19.66*	-333.2	*16.00*
		S	-257.68	*18.82*	-280	*20.36*	-328.5	*18.30*	-358.07	*22.24*

*AR, all-repeat; MR, mixed-repeat; S, switch.*

**Table 3:** Correlation between peak amplitude of stimulus-/response-locked P3 and age/model parameters for (A) each trial type and (B) mixing/switch cost on both ERP and model parameters.

A	Age	A	*A (age)*	V	*V (age)*	Ter	*Ter (age)*	Stepwise	*R*^2^ adjusted
**sP3 PA**				
All-repeat		-0.272		0.260				-	–
Mixed-repeat	-0.202	-0.251		0.238		-**0.305#**	-*0.237*	Ter	0.084
Switch	-0.242	-**0.327#**	-*0.261*	**0.382#**	***0.329#***	-0.261		V	0.137
**rP3 PA**					
All-repeat		-**0.405#**	-***0.419#***	**0.332#**	***0.328#***	-0.277		A	0.155
Mixed-repeat	-**0.286#**	-**0.413#**	-***0.321#***	**0.440#**	***0.389#***	-**0.431#**	-***0.340#***	V, Ter	0.304
Switch	-0.237	-**0.370#**	**-*0.294#***	**0.514#**	***0.471#***	-**0.399#**	-***0.311#***	V, Ter	0.341
**B**	**Age**	**A cost**	***A cost (age)***	**V cost**	***V cost (age)***	**Ter cost**	***Ter cost (age)***	**Stepwise**	***R*^2^ adjusted**
**sP3 PA**					
Mixing cost	**0.296#**							age	0.078
Switch cost				0.202				–	–
**rP3 PA**					
Mixing cost	**0.294#**	0.214				0.244		age	0.078
Switch cost		0.208		**0.309#**	*0.283*	-0.216		V	0.085

*Partial correlations when controlling for age are shown in italics. Significant *r*-values are marked # and *r* values that did not survive correction are also shown to illustrate where a similar pattern is evident across trial types. Positive correlations relate to larger amplitude or later change in amplitude. Variables selected in stepwise linear regression model and associated *R*^23^ adjusted are also shown.*

## RESULTS

### RT AND MODEL PARAMETERS^5^

**Figure 2** shows RT, and model parameters presented for all-repeat, mixed-repeat and switch trials across the age range. RT mixing cost and switch cost effects varied in size with age (**Figure 2A**). Specifically, mixing cost increased quadratically across the adult lifespan, whereas switch cost showed a small but significant linear reduction. Both effects were due to a disproportionate increase in RT for mixed-repeat than for switch and all-repeat trials in older adults.

Model parameters are shown in **Figures 2B–D**. Non-decision time increased linearly for all trial types with age. Younger adults showed no Ter difference between trial types, but switch trial Ter was differentially larger in older adults. Younger adults had a higher response criterion on switch followed by mixed-repeat and all-repeat trials. With increasing age, criterion disproportionately increased for mixed-repeat trials, resulting in no significant criterion difference between switch and mixed-repeat trials in older adults. Drift rate reduced with increasing trial type difficulty and with increasing age. The rate of the age effect was disproportionately larger for mixed-repeat trials, resulting in no significant difference between switch and mixed-repeat trials in the older age range for drift rate.

### ERP DATA

**FIGURE 3:**
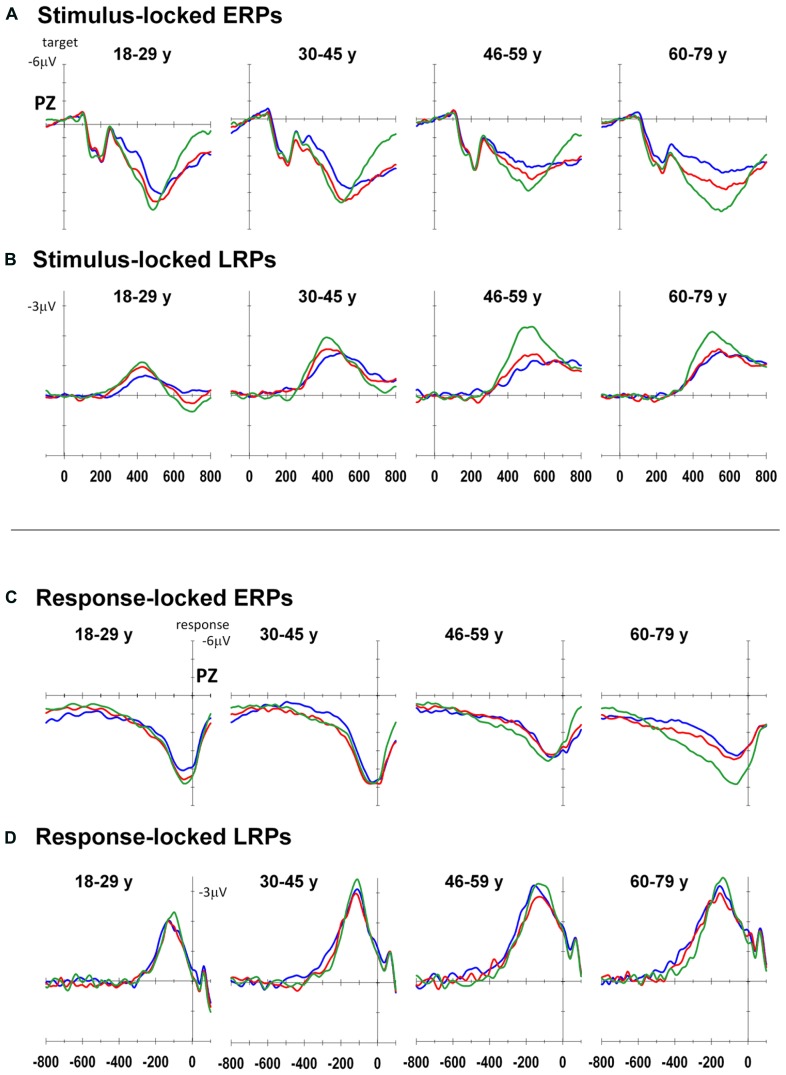
**Event-related potentials for each age group: Stimulus-locked ERP (A) and LRP (B), response-locked ERP (C) and LRP (D) for all-repeat (green), mixed-repeat (red), and switch (blue) trials**.

**Figure 3** shows stimulus-locked and response-locked ERP waveforms at Pz as well as stimulus-locked and response-locked LRP waveforms at C3–C4 for both groups. **Table 2** shows group means for stimulus-locked and rP3 and LRP waveforms.

#### Stimulus-locked vs. response-locked P3

Stimulus-locked P3 peaked earlier for mixed-repeat than switch trials [switch cost: *F*(1,92) = 5.49, *p* = 0.021; **Figure 3A**]. It also peaked earlier for all-repeat than mixed-repeat trials, but not uniformly across age groups [age × mixing cost: *F*(1,92) = 12.47, *p* = 0.001; **Table 2**]. The large mixing effect was significant in Young but not Old adults [*F*(1,24) = 17.84, *p* < 0.001; *p* > 0.30, respectively].

Peak sP3 amplitude was larger for mixed-repeat than switch trials [*F*(1,92) = 27.05, *p* < 0.001]. A mixing cost effect emerged with increasing age [age × mixing: *F*(1,92) = 9.63, *p* = 0.003; age2 × mixing: *F*(1,92) = 4.09, *p* = 0.046]. As shown in **Figure 3A**, sP3 amplitude did not differ between the two types of repeat trials in the younger age range, but was disproportionately smaller for mixed-repeat trials in older adults, resulting in a large mixing effect on the sP3. Direct group comparisons confirmed that a mixing effect on sP3 peak amplitude was present in Old but not Young adults [*F*(1,29) = 16.59, *p* < 0.001; *p* > 0.23, respectively].

For both trial types in the mixed-task block, rP3 peak amplitude reduced linearly with age [*F*(1,92) = 4.92, *p* = 0.029; see **Table 2**], but there was no difference in rP3 amplitude between switch and mixed-repeat trials (*F* < 1; **Figure 3C**). There was a large age effect on mixing cost [age × mixing: *F*(1,92) = 10.14, *p* = 0.002; age2 × mixing: *F*(1,92) = 8.99, *p* = 0.003]. As shown in **Figure 3C**, the younger age ranges showed little mixing effect on rP3 peak amplitude, whereas the older age range showed a large differentiation between mixed-repeat and all-repeat trials. Group analyses confirmed a highly significant mixing cost on rP3 amplitude in Old but a marginal effect in Young adults [2.8μV, *F*(1,29) = 22.59, *p* < 0.001 vs. 0.96μV, *F*(1,24) = 4.57, *p* = 0.043, respectively; **Table 2**].

In summary, there was a large switch effect on sP3 peak latency and peak amplitude, which was not evident for rP3 peak amplitude. This is consistent with sustained stimulus-level interference on switch relative to mixed-repeat trials across the entire adult age range despite the fact that participants were highly prepared and practiced. The large mixing effect on sP3 peak amplitude seen in older adults was also evident in rP3 peak amplitude, indicating that older adults experienced sustained response-level interference for mixed-repeat relative to all-repeat trials.

#### Relationship between P3 measures and model parameters

Larger sP3 amplitude was associated with faster performance as indexed by a less conservative criterion, higher drift rate and faster non-decision time (**Table 3A**). These relationships were evident across most trial types but were stronger for switch trials. All model parameters showed stronger correlations with rP3 than sP3 amplitude and these remained significant when controlling for age. As predicted, the relationship between Ter and sP3 amplitude was eliminated when controlling for rP3 amplitude (*p* > 0.30). Switch cost showed a weak correlation between drift rate and both sP3 and rP3 amplitude and the latter remained significant when controlling for age (**Table 3B**). Together these findings indicate that individual variability in rP3 amplitude for each of the three trial types is associated with faster performance mediated by all three model parameters. However, independent of age, a greater reduction in rP3 amplitude for switch vs. mixed-repeat trials is associated with residual switch cost on drift rate, consistent with greater difficulty with decision-related processes on switch trials. Age was moderately correlated with residual mixing cost on both sP3 and rP3 amplitude (**Table 3B**), consistent with a larger mixing effect on sP3 and rP3 amplitude emerging only in older adults.

Stepwise linear regression with each ERP measure as the outcome variable and model parameters and age as predictors confirmed the above pattern of results. Drift rate was the strongest predictor of rP3 amplitude for both trials in mixed-task blocks, and together with Ter predicted up to 34% of the variance in rP3 amplitude (**Table 3**). However, switch cost on drift rate was only a weak predictor of switch cost on rP3 amplitude.

#### Stimulus-locked and response-locked LRP

The onset latency of the sLRP increased approximately 60 ms across the age range [age: *F*(1,86) = 26.60, *p* < 0.001; **Figure 3B**]. sLRP latency did not differ between all-repeat and mixed-repeat trials (*p* > 0.10). However, sLRP onset was delayed for switch relative to mixed-repeat trials in the younger but not older range of the age scale [age × switch: *F*(1,86) = 5.47 *p* = 0.022]. This was confirmed by a significant group × switch interaction [*F*(1,49) = 5.55, *p* = 0.022], indicating that sLRP emerged earlier for mixed-repeat trials relative to switch trials in young adults, whereas old adults showed delayed sLRP onset for all three trial types.

The response-locked LRP (rLRP) emerged earlier with increasing age [*F*(1,90) = 33.93, *p* < 0.001], with rLRP duration being approximately 100 ms longer in the Old relative to the Young group [*F*(1,51) = 40.51, *p* < 0.001; **Figure 3D**]. rLRP duration was longer for mixed-repeat than all-repeat trials [mixing: *F*(1,90) = 4.93, *p* = 0.029], but group comparisons showed that this mixing effect was significant for the Old but not the Young group [*F*(1,28) = 10.74, *p* = 0.003; *p* > 0.12, respectively]. rLRP onset latency did not differ between mixed-repeat and switch trials (*F* < 1). So, rLRP duration increased with age, and this effect was larger for mixed-repeat trials in older adults.

#### Relationship between LRP measures and model parameters

Earlier onset of the sLRP was moderately correlated with faster performance as indicated by lower criterion, higher drift rate and shorter non-decision time (**Table 4A**). These relationships were largely restricted to the two repeat trial types and retained when controlling for age. In addition, there were significant correlations between mixing cost on sLRP onset latency and mixing cost on criterion and drift rate, and these relationships were also preserved when controlling for age (**Table 4B**). So, greater delay in sLRP onset for mixed-repeat relative to all-repeat trials was associated with slower performance for the more difficult mixed-repeat trials. Linear regression showed that criterion was the strongest predictor of mixing cost variability in sLRP onset. So, consistent with higher criterion resulting in slower decision and later response selection, adjusting criterion higher for mixed-repeat relative to all-repeat trials resulted in later onset of sLRP for those trials. Interestingly, while age was significantly correlated with sLRP onset latency for all trial types, it did not correlate with mixing cost on this measure, nor did it moderate the relationship between mixing cost effects on sLRP onset and response criterion (**Table 4**). These relationships were only weak for switch trials and switch cost.

**Table 4:** Correlation between onset latency of stimulus-/response-locked LRP and age/model parameters for (A) each trial type and (B) mixing/switch cost on both LRP and model parameters.

A	Age	A	*A (age)*	V	*V (age)*	Ter	*Ter (age)*	Stepwise	*R*^2^ adjusted
**sLRP onset**					
All-repeat	**0.505#**	**0.457#**	***0.299#***	-**0.448#**	-***0.345#***	**0.493#**	***0.288#***	age, V, Ter	0.382
Mixed-repeat	**0.434#**	**0.474#**	***0.346#***	-**0.405#**	-***0.362#***	0.281		A	0.216
Switch		0.229	*0.210*					–	–
**rLRP onset**					
All-repeat	-**0.389#**	-**0.561#**	-***0.471#***	**0.427#**	***0.346#***	-**0.689#**	-***0.618#***	Ter, A	0.588
Mixed-repeat	-**0.350#**	-**0.289#**				-**0.333#**		age	0.113
Switch	-**0.372#**	-0.275		0.200		-**0.481#**	-***0.383#***	Ter	0.223
**B**	**Age**	**A cost**	***A cost (age)***	**V cost**	***V cost (age)***	**Ter cost**	***Ter cost (age)***	**Stepwise**	***R*^2^ adjusted**
**sLRP onset**					
Mixing cost		**0.440#**	***0.483#***	**0.380#**	***0.381#***	-0.240		A	0.184
Switch cost	-0.244	0.244				-0.250		–	–
**rLRP onset**					
Mixing cost		0.235						–	–
Switch cost								–	

*Partial correlations when controlling for age are shown in italics. Significant *r*-values are marked # and *r* values that did not survive correction are also shown to illustrate where a similar pattern is evident across trial types. Positive correlations relate to larger amplitude or later change in amplitude. Variables selected in stepwise linear regression model and associated *R*^*2*^ adjusted are also shown.*

The duration of the rLRP was moderately to strongly correlated with all model parameters for all-repeat trials (**Table 4A**) and these correlations were retained when controlling for age. Ter was the strongest predictor of rLRP onset latency for these trial types followed by criterion. For mixed-repeat trials, significant but weaker correlations were found between rLRP onset latency and both Ter and criterion, and these were eliminated when controlling for age. Ter was the only parameter to be associated with rLRP onset for switch trials. In fact, linear regression showed that age was the only predictor of rLRP onset for mixed-repeat trials.

## DISCUSSION

### POST-TARGET PROCESSES CONTRIBUTING TO RESIDUAL MIXING COST AND RESIDUAL SWITCH COST

Karayanidis et al. (2011) showed that highly prepared participants show sustained residual mixing and switch costs even after substantial task practice, and that these RT costs arise from decision processes. That is, increasing trial difficulty (all-repeat vs. mixed-repeat vs. switch) was associated with a progressive increase in response criterion and decrease in drift rate, but not non-decision time. In this study, we sought to identify whether stimulus-level or response-level interference contributed to these residual mixing and switch costs.

Electrophysiologically, the residual mixing cost was represented in delayed peak latency of the sP3 for mixed-repeat than for all-repeat trials (e.g., mean delay of 35 ms in Young group). In contrast, younger adults showed no mixing effect on the amplitude of either the stimulus-locked or the rP3, or on the latency of stimulus-locked or response-locked LRP (see in **Figure 3**). Together with the mixing effect on the amplitude of the earlier frontal N2 (see Figure 8 from Karayanidis et al., 2011), this pattern of findings suggests that residual mixing cost represents stimulus-level interference on mixed-repeat trials, resulting in slower decision processes. This is consistent a more conservative response criterion and slower drift rate on these trials (**Figure 2D**), resulting in larger RT mixing cost. Thus, incongruently mapped bivalent stimuli continue to elicit interference at the level of stimulus; but processes involved in stimulus-response mapping, response selection and response programming benefit equally from task repetition for both types of repeat trials. Hence, in young adults, the residual mixing cost for these bivalent stimuli results from a greater difficulty with attending to the relevant stimulus dimension or inhibiting interference from the irrelevant stimulus dimension on mixed-repeat than all-repeat trials.

Within the mixed-task block, younger adults flexibly adjusted response criterion on a trial-by-trial basis so as to maintain a more cautious criterion on the more difficult switch trials and a more liberal one for the easier mixed-repeat trials (see also Karayanidis et al., 2009; Mansfield et al., 2011). They also showed a slower drift rate for switch trials, resulting in a residual RT switch cost. Electrophysiologically, switch trials were associated with a smaller and later sP3 across the entire age range (**Figure 3A**). In younger adults, switch cost also affected sLRP onset latency, but there was no significant effect on either the rP3 or the response-locked LRP. These findings suggest that bivalent, incongruently-mapped stimuli produce greater interference for switch trials than for mixed-repeat trials (see also Gajewski et al., 2010). For switch trials, this interference affects not only stimulus selection and evaluation (see also N2 effects in Karayanidis et al., 2011), but also response selection, as represented by the delay in sLRP onset latency. However, despite using tasks with bivalent stimuli and incongruent stimulus pairs, residual mixing and switch costs were not evident in rP3 amplitude or rLRP onset latency, which represent decision-response mapping and response programming, respectively.

### PROCESSES CONTRIBUTING TO INCREASED RESIDUAL MIXING COST IN OLDER ADULTS

This study also examines the locus of the large age-related increase in post-stimulus interference, as indicated by the sustained residual mixing cost and the emergence of a large mixing effect on the sP3 in the oldest age group (Karayanidis et al., 2011). We examined whether the mixing effect on P3b amplitude in older adults is related to sustained post-stimulus interference affecting stimulus-related or response-related processes for repeat trials in a mixed-task block.

The large mixing cost effect on sP3 amplitude seen in the oldest group was maintained in the rP3. In addition, age was significantly correlated with mixing cost on both sP3 and rP3 amplitude, and the relationship between mixing cost on sP3 amplitude and age was eliminated when controlling for rP3 amplitude. These findings indicate that the age-related increase in mixing cost on P3 amplitude represents a specific difficulty in post-decision processes that map a decision to the corresponding response (Verleger et al., 2005).

Consistent with this, older adults also showed a significant mixing cost on rLRP onset latency. Longer interval between rLRP onset and response indicates slower response programming or activation (Osman et al., 1995; Leuthold and Jentzsch, 2002). In task-switching, earlier rLRP onset latency has been previously found for switch vs. mixed-repeat trials in people with schizophrenia, where it was interpreted as suggestive of greater equivocation in response activation for the more difficult switch trials (Karayanidis et al., 2006). In the current context, the oldest group showed a large mixing effect on rP3 amplitude and rLRP duration, but no switching effect on either of these measures. Furthermore, although the young group showed delayed sLRP onset for switch vs. other trials, the old group showed no trial type effect on sLRP onset. These findings suggest that the large increase in RT mixing cost in old adults that persists despite substantial task practice and greater preparation for mixed-repeat trials is likely to arise from sustained response-level interference for both trial types in the mixed-task block. The fact that these mixing effects in old adults occurred without concurrent switch effects is consistent with earlier evidence that older adults do not efficiently differentiate between switch and mixed-repeat trials.

Age effects on residual switch cost also point to less efficient processing of mixed-repeat trials. In the Introduction we argued that, as older adults set a similar response criterion and prepare similarly for both switch and mixed-repeat trials, they might be expected to treat these two trials similarly after stimulus onset. In fact, like younger participants, older adults showed a smaller P3 for switch than mixed-repeat in stimulus-locked but not response-locked waveforms. However, unlike younger participants, older adults showed no delay in sLRP onset for switch relative to mixed-repeat trials. At face value, this pattern of switch effects on sP3 vs. sLRP seen in older adults appears counterintuitive. It suggests that any differential stimulus-driven interference on switch trials has less impact on response selection processes in older than in younger people. However, the fact that older participants showed sustained differences between all-repeat and mixed-repeat trials across most measures suggests that, in fact, the absence of a switch effect on sLRP is not due to *more efficient* response selection for switch trials but to *less efficient* response selection for mixed-repeat trials compared to all-repeat trials.

Together these findings suggest that, unlike younger adults, older adults do not show a repetition priming benefit on response-related processes for mixed-repeat trials. This is broadly consistent with previous evidence that older adults benefit less from priming in both task-switching (Cepeda et al., 2001) and repetition priming (e.g., Karayanidis et al., 1993) paradigms.

## CONCLUSION

In conclusion, these findings show that residual mixing cost arises from stimulus-level interference, whereas residual switch cost arises from post-stimulus interference affecting both stimulus evaluation and response selection. Our highly practiced and healthy older adults showed no task repetition benefit on post-stimulus processes associated with decision-response mapping, response selection and programming. This finding helps explain both the higher mixing cost and the smaller switch cost in older as compared to younger adults. Specifically, it is reasonable to argue that *because* older adults do not benefit from task repetition, they apply greater proactive control for these trial types, strategically preparing for them as if they were switch trials in order to compensate for less efficient automatic priming of response-related processes. This interpretation implies that structural changes in the efficiency of more “automatic” fast networks with increasing age (Forstmann et al., 2011) may result in a strategic adjustment of cautiousness for easier conditions and hence more reliance on slower networks that emphasize accuracy over speed. Critically, this interpretation suggests that the setting of a similar response criterion for mixed-repeat and switch trials may not due to an inability to flexibly adjust criterion on a trial by trial basis, but rather to a strategic decision to compensate for the lack of task repetition benefit on mixed-repeat trials. As task repetition benefit on response processes is likely to be reduced when using bivalent as compared to univalent response-sets, it should be possible to test this prediction by examining whether older participants adjust response criterion differentially for switch and mixed-repeat trials for univalent but not bivalent task-sets. This should help determine whether the age-related changes in performance reflect an active strategy that is selected by older adults to overcome low level processing deficits or whether structural changes in higher level processing that limits flexible adjustment.

Our findings are consistent with the abundant evidence that older participants show preference for accuracy-based strategies (Ratcliff et al., 2005, 2006a,b) and provide insight into potential reasons for such a preference. They are also consistent with evidence suggesting that older participants apply greater processing resources for the completion of a range of cognitive tasks (Cabeza, 2002; Dennis and Cabeza, 2008) and with models of cognitive aging that posit the adoption of compensatory strategies to overcome decline in low level sensory and motor processes (e.g., Stern, 2002; Park and Reuter-Lorenz, 2009; Vallesi et al., 2011).

## Conflict of Interest Statement

The authors declare that the research was conducted in the absence of any commercial or financial relationships that could be construed as a potential conflict of interest.

## Footnotes

1 Some studies estimate mixing cost as the difference between single-task and mixed-task trials, a measure others refer to as “general switch cost.” Here we consistently refer to mixing cost as defined in the text, regardless of the definition used in each study reviewed.

2 Note that Adrover-Roig and Barceló (2010) and Eppinger et al. (2007) showed larger stimulus-locked ERP differentiation between switch and mixed-repeat trials in old than in young adults. However, both task requirements and amount of practice differ considerably from the Karayanidis et al. (2011) paradigm.

3 As there were large age main effects on sP3 peak amplitude, all analyses were confirmed using ratio scores calculated in relation to the average peak sP3 amplitude for each individual.

4 Results were confirmed using onset latency measured using 50% of peak amplitude and the regression based 1df method (Mordkoff and Gianaros, 2000).

5 These results were presented in detail in Karayanidis et al. (2011). Here we provide a brief overview of findings to support presentation of stimulus- and response-locked waveform analysis.
